# Technical Note: A step‐by‐step guide to Temporally Feathered Radiation Therapy planning for head and neck cancer

**DOI:** 10.1002/acm2.12893

**Published:** 2020-05-08

**Authors:** Shireen Parsai, Richard L. J. Qiu, Peng Qi, Juan C. L. Alfonso, Jeremy Donaghue, Eric Murray, David Majkszak, Nicole Dorio, Clifton D. Fuller, Kristy Brock, Shlomo Koyfman, Neil Woody, Nikhil Joshi, Jacob G. Scott

**Affiliations:** ^1^ Department of Radiation Oncology Taussig Cancer Institute Cleveland Clinic Cleveland OH USA; ^2^ Department of Radiation Oncology Winship Cancer Institute of Emory University Atlanta GA USA; ^3^ Braunschweig Integrated Centre of Systems Biology Hemholtz Centre of Infection Research Braunschweig Germany; ^4^ Department of Radiation Oncology MD Anderson Cancer Center Houston TX USA

**Keywords:** head and neck planning, IMRT, reduce toxicity, temporally feathered radiation therapy, TFRT

## Abstract

**Purpose:**

Prior *in silico* simulations propose that Temporally Feathered Radiation Therapy (TFRT) may reduce toxicity related to head and neck radiation therapy. In this study we demonstrate a step‐by‐step guide to TFRT planning with modern treatment planning systems.

**Methods:**

One patient with oropharyngeal cancer planned for definitive radiation therapy using intensity‐modulated radiation therapy (IMRT) techniques was replanned using the TFRT technique. Five organs at risk (OAR) were identified to be feathered. A “base plan” was first created based on desired planning target volumes (PTV) coverage, plan conformality, and OAR constraints. The base plan was then re‐optimized by modifying planning objectives, to generate five subplans. All beams from each subplan were imported onto one trial to create the composite TFRT plan. The composite TFRT plan was directly compared with the non‐TFRT IMRT plan. During plan assessment, the composite TFRT was first evaluated followed by each subplan to meet preset compliance criteria.

**Results:**

The following organs were feathered: oral cavity, right submandibular gland, left submandibular gland, supraglottis, and OAR Pharynx. Prescription dose PTV coverage (>95%) was met in each subplan and the composite TFRT plan. Expected small variations in dose were observed among the plans. The percent variation between the high fractional dose and average low fractional dose was 29%, 28%, 24%, 19%, and 10% for the oral cavity, right submandibular, left submandibular, supraglottis, and OAR pharynx nonoverlapping with the PTV.

**Conclusions:**

Temporally Feathered Radiation Therapy planning is possible with modern treatment planning systems. Modest dosimetric changes are observed with TFRT planning compared with non‐TFRT IMRT planning. We await the results of the current prospective trial to seeking to demonstrate the feasibility of TFRT in the modern clinical workflow (NCT03768856). Further studies will be required to demonstrate the potential benefit of TFRT over non‐TFRT IMRT Planning.

**Abbreviations**
IMRT: intensity modulated radiation therapy
OAR: organ at risk
TFRT: temporally feathered radiation therapy

## 1 INTRODUCTION

Head and neck squamous cell carcinomas are now primarily treated with definitive, nonsurgical, organ sparing approaches with radiotherapy. Since the advent of intensity‐modulated radiation therapy (IMRT), acute and late toxicities of radiotherapy have declined dramatically but still remain prevalent. In a recent study of patients receiving definitive radiotherapy with or without chemotherapy for oropharyngeal cancer, 42% of patients experienced acute grade 3 or greater toxicity.^1^ In effort to reduce toxicity, Temporally Feathered Radiation Therapy (TFRT) has been introduced as a novel planning technique. Prior *in silico* simulations of TFRT have demonstrated potential for reduced normal tissue toxicity compared with non‐TFRT IMRT technique.^2^

**Fig. 1:**
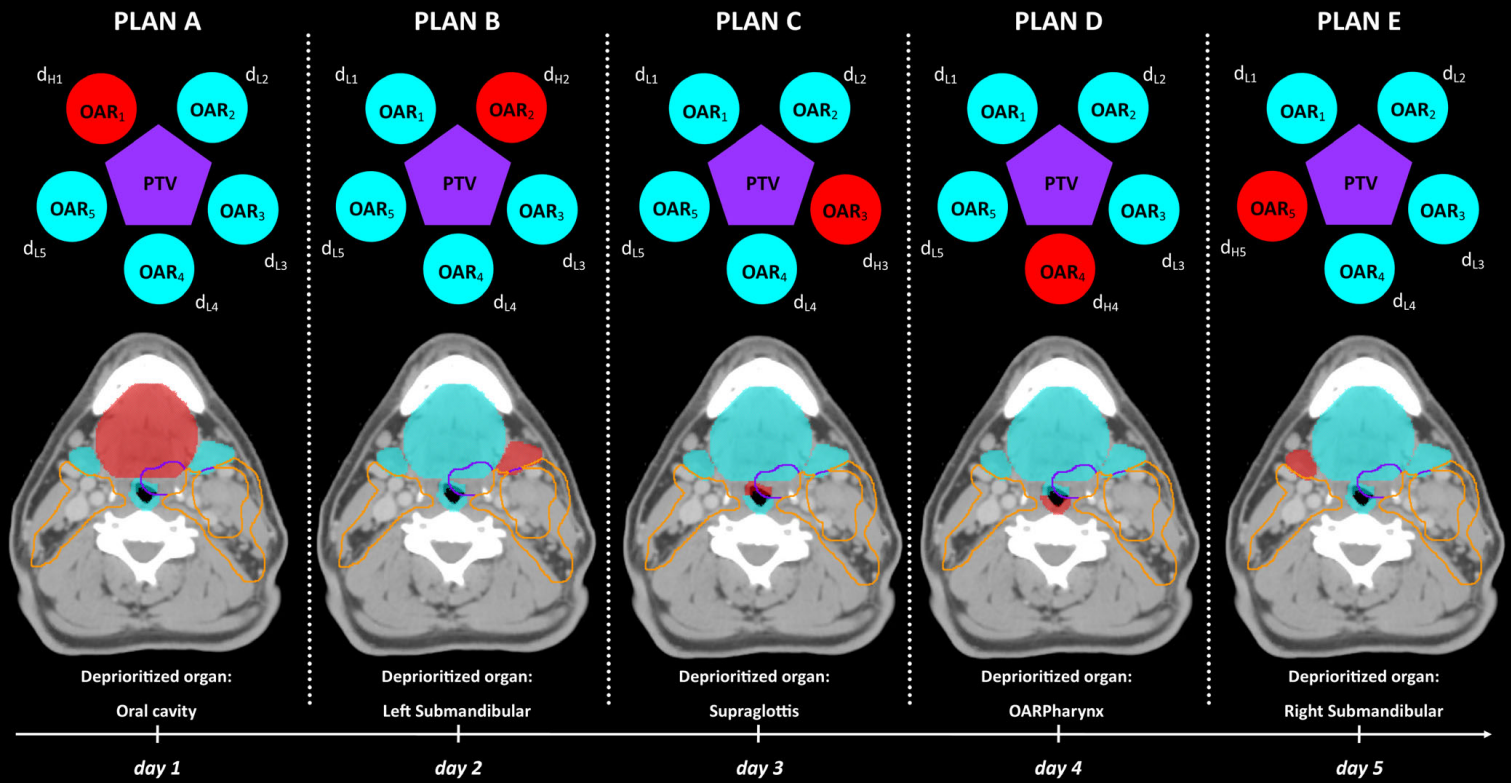
Schematic representation of Temporally Feathered Radiation Therapy. The target volume (purple pentagon) is surrounded by five organs at risk (circles). Five individual radiation plans are created for each day of the week whereby a higher fractional dose, d_H_ (red), is delivered to the deprioritized OAR of interest and the four prioritzed OARs receive a lower fractional dose, d_L_ (blue)

In non‐TFRT IMRT planning, a daily fractional dose is delivered to the target and surrounding organs at risk (OAR) with each fraction of radiotherapy. In contrast, TFRT planning varies the dose delivered to the surrounding OARs, while keeping the dose to the target unchanged (i.e., the target volume should be covered by > 95% of the prescribed dose). TFRT plans are composed of five isocurative (same tumor dose) *subplans, each of* which are delivered once per week as illustrated in Fig. 1. In each subplan, one OAR is deprioritized and therefore receives a higher fractional dose (d_H_). By pushing dose into the deprioritized organ, the other OARs of interest will receive a lower fractional dose (d_L_). Resultantly, each feathered OAR will receive a slightly higher fractional dose once weekly, followed by slightly lower fractional doses the remaining four fractions. Previously it has been hypothesized that if a single fraction of d_H_ is delivered to an OAR once weekly and four fractions of d_L_ on the remaining days, normal tissues may exhibit increased recovery despite higher total doses delivered to the OAR. This increase in normal tissue recover may reduce clinical toxicity.^2^ This technique seeks to optimize normal tissue recovery through the nonlinear temporal nature of healing.

In this study, we describe the step‐by‐step planning process by which temporally feathered radiation therapy plans are generated and assessed. We will also provide a dosimetric analysis of the TFRT plan referenced against a non‐TFRT IMRT plan.

## 2 MATERIALS AND METHODS

One patient with squamous cell carcinoma of the oropharynx planned for definitive radiation therapy (with concurrent chemotherapy) using IMRT techniques was replanned using the TFRT technique. The prescription dose was 70 Gy/ 35 fractions to the high‐dose planning target volumes (PTV) and 56 Gy/ 35 fx to the low‐dose PTV using simultaneous integrated boost technique. The TFRT technique was evaluated for technical feasibility and was also compared dosimetrically to the non‐TFRT IMRT plan.

### 2.A Simulation and target volume definition

The patient was immobilized with a 5‐point mask (Orfit, Belgium) and a customized cushion. A bite block covered with dental wax was used to create separation between the tongue and the hard palate. Intravenous contrast was administered. The treatment planning CT was obtained in 3 mm slices using Philips CT Big Bore simulator.

The physician was responsible for delineating the gross tumor volumes (GTV), clinical target volumes (CTV), and PTV. To delineate the tumor volumes, diagnostic images were coregistered with the treatment planning CT. The PTV margin was 2.5 mm. All organs at risk (OARs) were also delineated by the treating physician.

### 2.B FRT plan generation

All treatment plans were generated using Pinnacle^3^ treatment planning software (version 9.10, Philips Healthcare, Fitchburg, WI, USA). Before TFRT planning, the treating physician and a physicist identified five OARs to be feathered based on proximity to the target. These OARs may have included but were not limited to the oral cavity, each submandibular gland, each parotid gland, OAR pharynx, supraglottis, larynx, and esophagus. Five isocurative subplans (same tumor dose) were generated in which one OAR was deprioritized during planning. In an index subplan, the deprioritized organ receives a higher fractional dose (d_H_) compared to the fractional dose delivered in the composite TFRT plan. The remaining four OARs chosen to be feathered would receive a lower dose (d_L_) than the fractional dose delivered in the composite TFRT plan. Each subplan is scheduled to be delivered a specific day of the week. This allows each OAR of interest to receive d_H_ once weekly and d_L_ the remaining 4 days of the week. The dose delivered to the PTV is not altered. The prescription dose must encompass 95% of each PTV volume (i.e., PTV_7000 and PTV_5600). Organs at risk nonoverlapping with the PTV volume were parameterized for feathering. The max point dose for d_H_ delivered to an OAR nonoverlapping with PTV must comply with: 0.03 cc of the deprioritized organ cannot exceed >110% of the prescription dose.

Two 6 MV VMAT full‐arc beams (182⁰–178⁰) with different collimator angles (10⁰ and 350⁰) were used. A 0.064 cc dose grid resolution was chosen. The dose calculation grid covered all the planning structures. The dose calculation algorithm applied was adaptive convolution. Maximum iterations number was 40 and convolution dose iteration was set to 15.

**Fig. 2:**
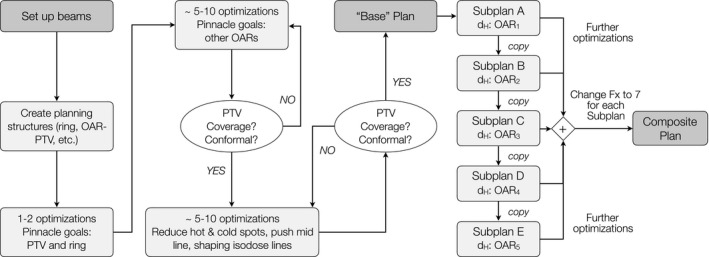
Planning flowchart to develop five TFRT subplans and the final composite plan. First, planning structures such as normal tissue rings, OAR‐PTV, mid‐line structures, etc., were created. A good “base” plan, which does not push on the five TFRT feathering target OARs, was generated before making the five TFRT subplans. Then, planning goals for OARs B‐E were added to the planning objectives. Further optimizations were run to create subplan A from the base plan. Subplan B, C, D, and E were created by copying the previous subplan and optimizing after modifying prioritized and deprioritized OARs. Further improvements were made to each subplan to meet the planning criteria. At the end, a composite plan was made by importing all the beams from the subplans into one trial. The fraction number of each prescription was changed to 7, for a total of 35 fractions for the composite plan

The workflow for TFRT treatment planning is demonstrated in Fig. 2. First, the planning structures were generated and reviewed. For the low‐dose PTV (i.e., PTV_5600), a planning PTV_5600_obj structure was created by subtracting the 5 mm expansion of the PTV_7000 from the original PTV_5600, to account for permissible dose fall‐off from the high‐dose PTV_7000. Two ring structures were created to aid in confining the spread of 50% and 30% of the prescription dose (i.e., 70 Gy). These ring structures were created by subtracting the combined PTV expansion volumes (1 cm expansion and 3 cm expansion) and all the five feathered OARs from the external contour.

The “base plan” which is later manipulated to create each subplan is generated through the following steps. First, a limited set of structures which include the PTVs and ring structures are fed into the optimizer. Then, optimization rounds were completed adding 3–5 normal structure objectives (not including the feathers OARs) into the optimizer. With each optimization round, the PTV coverage, plan conformality, and OAR constraints were evaluated. Lastly, additional planning structures were created manually to reduce hot and cold spots within the PTV volumes, push dose away from midline structures, remove low‐dose spillage, and shape the isodose lines.

To generate a TFRT subplan, the base plan was re‐optimized with planning objectives for the four prioritized OARs (i.e., those receiving d_L_). No planning objectives were added to the optimizer for the deprioritized OAR (i.e., the OAR receiving d_H_ in that particular subplan). Five subplans were generated from each base plan. The five subplans were used to create the composite TFRT plan. Each subplan contributed one fifth of the total dose.

### 2.C TFRT plan assessment

Plan assessment and approval were performed by the physician following a meticulous protocol. First the composite TFRT plan was reviewed, then each subplan was individually reviewed. Both the composite plan and each TFRT subplan met the criteria detailed in the supplementary material. Normal tissue constraints were adapted from RTOG 1016 (NCT01302834). As per institutional protocol, a “scorecard” was generated for the composite plan and filed in Mosaiq indicating that safety metrics were met.

### 2.D Quality assurance

Standard quality assurance procedures were followed per AAPM TG‐218.^3^ Each subplan was quality assured separately. The patient‐specific QA for each TFRT subplan was generated and recorded. Both the physicist and the attending physician reviewed and signed the QA document.

## 3 RESULTS

### 3.A TFRT plan overview

The following organs were feathered: oral cavity, right submandibular gland, left submandibular gland, supraglottis, and OAR Pharynx (posterior pharyngeal wall). Time required for creation, optimization, and evaluation of all TFRT subplans as well as the composite plan was 7 days.

### 3.B PTV coverage

Greater than 95% of each PTV was covered by the prescription dose in each subplan and the composite TFRT plan. Table 1 demonstrates the dosimetric constraints and data from the composite TFRT plan compared with the previously planned non‐TFRT IMRT plan. *OAR Dosimetric Differences*.

**Table 1:** Dosimetric comparison between IMRT plan and composite TFRT plan

Structure	Compliance criteria per protocol	Dose achieved in non‐TFRT IMRT plan	Dose achieved in Composite TFRT Plan
PTV_7000	>95% coverage with 70 Gy	95.22%	95.99%
PTV_5600	>95% coverage with 56 Gy	95.35%	96.66%
PTV_7000	D1cc ≤ 77 Gy	74.59 Gy	75.20 Gy
Conformity index for PTV_7000^a^	No constraints per protocol	1.06	1.11
Conformity index for PTV_7000^a^	No constraints per protocol	1.54	1.53
Brainstem	0.03 cc ≤ 60 Gy	12.20 Gy	12.00 Gy
Brainstem PRV	0.03 cc ≤ 63 Gy	13.20 Gy	15.14 Gy
Spinal cord	0.03 cc ≤ 45 Gy	28.78 Gy	28.26 Gy
Spinal cord PRV	0.03 cc ≤ 50 Gy	34.80 Gy	34.13 Gy
Lips	as low as achievable, goal mean < 20 Gy	5.89 Gy	6.32 Gy
Oral cavity	as low as achievable, goal mean < 30 Gy for uninvolved oral cavity	22.71 Gy	21.08 Gy
Right parotid	as low as achievable, goal mean < 26 Gy	12.90 Gy	12.03 Gy
Left parotid	as low as achievable, goal mean < 26 Gy	22.26 Gy	22.35 Gy
Right Submandibular	when not targeted, goal mean < 39 Gy	40.44 Gy	37.16 Gy
Left submandibular	when not targeted, goal mean < 39 Gy	42.76 Gy	43.50 Gy
OAR Pharynx	as low as achievable, goal mean < 45 Gy	40.85 Gy	43.77 Gy
Esophagus	as low as achievable, goal mean < 30 Gy	14.33 Gy	14.8 Gy
Supraglottis	as low as achievable, goal mean dose < 45 Gy	33.32 Gy	30.85 Gy
Larynx	as low as achievable, goal mean dose < 45 Gy	15.78 Gy	18.39 Gy

Abbreviations: IMRT, intensity‐modulated radiation therapy; OAR, organ at risk; TFRT, temporally feathered radiation therapy.

^a^ Conformity index calculated by dividing the prescription isodose volume by the planning target volume.

**Fig. 3:**
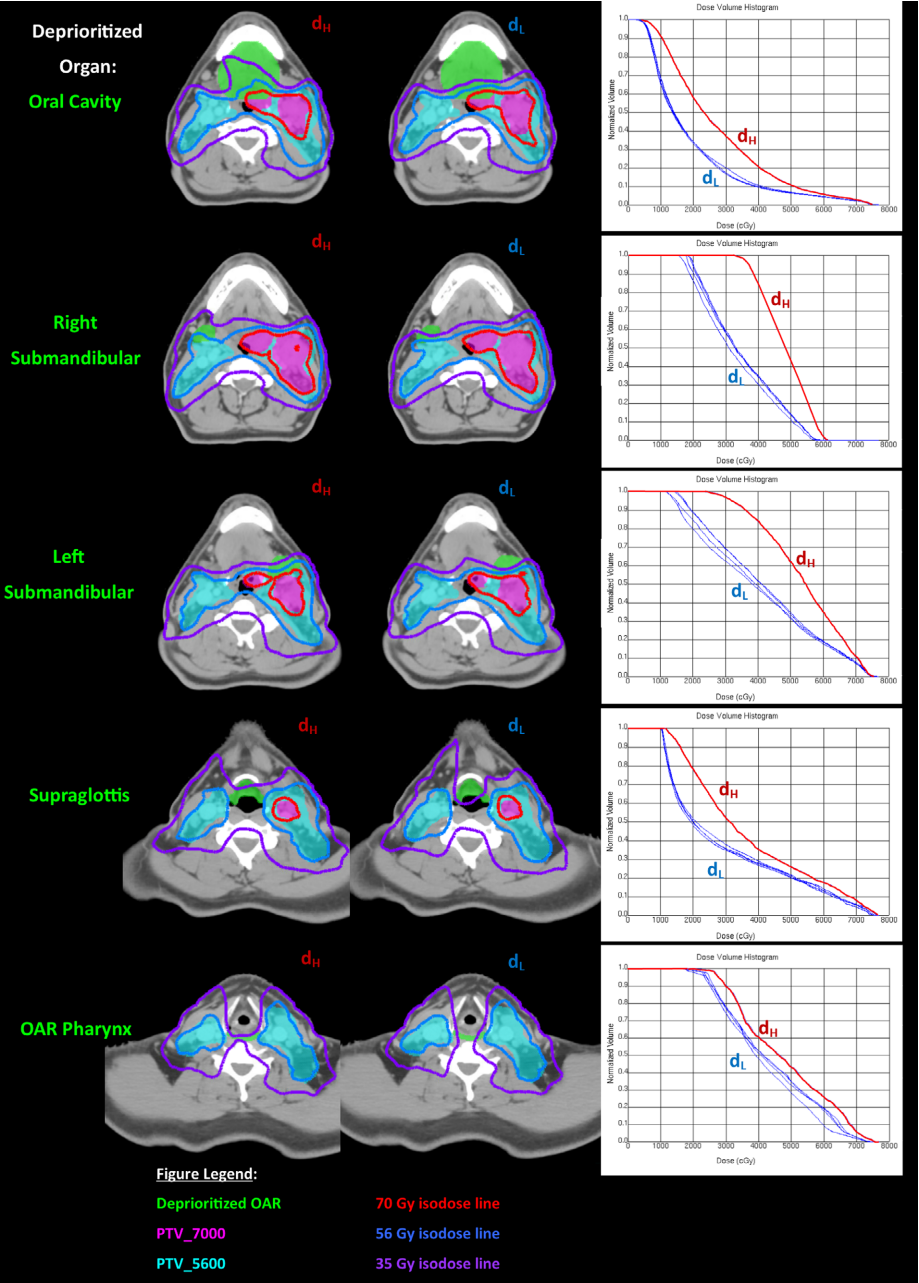
Representation of variation between d_H_ and d_L._ Axial slices with dose distributions are illustrated with associated dose volume histogram (DVH) for each feathered organ. The differences in dose distributions between the higher fractional dose (i.e., OAR deprioritized) and lower fractional dose can be observed (i.e., OAR constrained). The DVH demonstrates the dose to the OAR for each of five subplans

Table 2 demonstrates dosimetric differences in the dose delivered per fraction to each feathered organ with each subplan. The percent variation between the high fractional dose (d_H_) and average low fractional dose (d_L_) was 29%, 28%, 24%, 19%, and 10% for the oral cavity, right submandibular, left submandibular, supraglottis, and OAR pharynx nonoverlapping with the PTV. Figure 3 represents the physical dose distribution changes with each subplan for the feathered organs is represented in figure as well as the corresponding dose volume histogram changes.

**Table 2:** Daily fractional dose differences in each feathered OAR

Treatment plan	Fractional dose to ORAL_CAVITY (Gy)	Fractional dose to SUBMANDIBULAR_R (Gy)	Fractional dose SUBMANDIBULAR_L (Gy)	Fractional dose to SUPRAGLOTTIS (Gy)	Fractional dose to OAR PHARYNX (Gy)
Subplan A	**0.79**	0.94	1.15	0.84	1.18
Subplan B	0.57	**1.37**	1.13	0.84	1.24
Subplan C	0.56	0.99	**1.53**	0.85	1.23
Subplan D	0.55	1.01	1.20	**1.05**	1.23
Subplan E	0.55	1.00	1.20	0.86	**1.36**
Composite	0.60	1.06	1.24	0.88	1.25

Abbreviation: OAR, organ at risk.

Bold values represent the higher fractional dose, d_H_, for the deprioritized organ.

## 4 DISCUSSION

In this study, one patient previously treated for head and neck cancers was replanned using the temporally feathered radiation therapy technique. A step‐by‐step guide is detailed for radiation planning. Additionally, dosimetric data from the TFRT plan were compared against the previously planned IMRT plan.

The primary limitation of the TFRT technique is the time required for planning. Multiple techniques to reduce planning time will be reflected in future efforts. First, reducing computation time with treatment planning systems which utilize parallel computing will drastically reduce planning time. In our study, the majority of time required for plan generation in this study was due to computation time with the rate‐limiting step being creating the base plan. Subsequent modifications in the base plan to create subplans for each deprioritized organ require less planning time. The development of pinnacle scripts is expected to largely reduce the time required for generating a base plan and subsequent subplans. Aside from prolonged planning time, current practices demand quality assurance for each TFRT plan. We do not anticipate increased radiation delivery time. The proposed processes of radiation delivery are not modified, except for an added timeout to assure the correct ‘plan of the day’ is delivered (Appendix II).

Based on prior modeling, greater fluctuations between d_H_ and d_L_ will allow for the greatest reduction in NTCP. However, in practical application, increasing the percentage difference in dose between d_H_ and d_L_ may compromise the conformity of the plan. Importantly, during plan review the radiation oncologist should first review the composite TFRT plan. This is reflective of the entire treatment course and the physical dose distribution. In reviewing individual TFRT subplans, the understanding exists that any loss of conformity is also “feathered” between each fraction. Taking, for example, if increased posterior neck dose was marginally greater in subplan A, this may be less on the subsequent day when subplan B was delivered. Therefore, the physician should both evaluate the isodose lines on each individual subplan as well as the final composite TFRT plan together as a better indicator of the conformity of the plan. Each subplan and the composite TFRT plan must meet compliance criteria, and dose constraints as detailed in Appendix II.

In this study, all feathered organs were considered “parallel organs” and as such we used the simplified measure of dose, mean doses, as adapted from RTOG 1016. Each subplan and the composite TFRT were required to meet the compliance criteria and dose constraints set forth by the physician. There are ongoing efforts to define the best metrics to evaluate TFRT plans. In comparing a composite TFRT plan to a non‐TFRT IMRT plan, it was understood that though the dose to the organs at risk in the composite TFRT plan may have been greater than that of the non‐TFRT IMRT plan, it was previously hypothesized that the normal tissue complication probability may be less in the composite TFRT plan.^2^ This is owing to the assumed increased repair allowed in with the TFRT technique. Although once weekly a greater fractional dose was delivered to the deprioritized organ, the fractional dose was always less than 2 Gy per fraction. Therefore, we do not anticipate increased late toxicity. Notably, in this study we chose to feather five OARs for logistical reasons, however, this technique of planning can be implemented for any number of feathered organs.

Theoretically with TFRT, the five organs at risk (OAR) chosen to be feathered would receive a higher total dose. TFRT hypothesizes that despite receiving higher total doses, the feathered OARs would accumulate less toxicity due to improved time for normal tissue recovery.^2^ When TFRT subplans were generated clinically, not all feathered organs actually received a higher total dose. This was attributed to small subjectivity and variations that naturally occur in the planning and optimization process. The five organs feathered in this patient example included: oral cavity, right submandibular, left submandibular, supraglottis, and OAR Pharynx. The following organs received higher total doses in TFRT plans: left submandibular and OAR pharynx. Whereas the following organs received lower total doses in TFRT plans: oral cavity, right submandibular, and supraglottis. The organs not feathered which are standardly contoured and evaluated are shown in Table 1. In Table 1, it can be observed that large dosimetric differences in the other nonfeathered organs were not observed as intended. In other words, the dose was only fluctuated between the feathered organs.

Other researchers have also examined altering dose distributions over different fractions with the goal of reducing radiation‐related side effects.^4^, ^5^, ^6^, ^7^ However, the TFRT approach described in this study has two hallmark differences. First, Unkelbach et al. examined delivering hypofractionated radiotherapy to parts of the tumor, while maintaining a uniformly fractionated dose to the surrounding organs at risk.^5^ In contrast with TFRT planning, the dose to the target is not changed and rather only the dose to the organs at risk is modified. In this way, we are confident that the effects of radiotherapy with regards to tumor control are consistent with the currently established standards of care. Second, to account for the partially hypofractionated regimens, Unkelbach et al optimized plans based on BED. Liver tumors were used to model this treatment planning technique, and authors were able to demonstrate a 12–15% mean liver BED reduction compared to uniformly fractionated plans.^5^ Prior work on TFRT planning by Alfonso et al. quantified reduction in normal tissue toxicity by applying a dynamic NTCP model. Because the widely accepted linear quadratic model does not account for normal tissue recovery, a dynamic NTCP model was necessary to accurately model the interplay of TFRT, normal tissue recovery, and reduced toxicity.

Here, we present a step‐by‐step guide to TFRT planning using modern treatment planning systems. The currently open‐phase I feasibility trial (NCT03768856) seeks to evaluate feasibility implementing Temporally Feathered Radiation Therapy in the modern clinical workflow prospectively. As part of this study, the time required for treatment planning, plan evaluation, quality assurance, and delivery will be recorded.

## CONFLICTS OF INTEREST

The author has no conflict of interests to disclose.
