# Supplementary material for: Technical Note: A step‐by‐step guide to Temporally Feathered Radiation Therapy planning for head and neck cancer
Source: J Appl Clin Med Phys. 2020 May 8;21(7):209–15. doi: 10.1002/acm2.12893 (PMC7386183; doi:10.1002/acm2.12893)
Supplement: Supplementary file 1 [file ACM2-21-209-s001.docx]

Supplementary Material

**Appendix 1.** Compliance Criteria

|  | **Per Protocol** | **Variation Acceptable** | **Deviation Unacceptable** |
| --- | --- | --- | --- |
| Total RT dose to PTV_7000 (to 95% of the PTV) | 70 Gy | None | none |
| Minimum dose (“cold spot” within PTV_7000, not including portion near (<8 mm skin) defined for a point that is 0.03 cc in size | 66.5 Gy (equals 95% prescribed dose) | <66.5 but >63 Gy | ≤63 Gy |
| Maximum dose (“hot spot” >1 cc) within PTV_7000 | ≤77 Gy | >77 Gy but ≤82 Gy | >82 Gy |
|  |  |  |  |
| Total RT dose to PTV_5600 (to 95% of the PTV) | 56 Gy | ≥45 but <56 Gy | <45 Gy |
| Total RT dose to PTV_6300 (to 95% of the PTV) | 63 Gy    **Required when applicable** | ≥52 but <63 Gy | <52 Gy |
| Total RT dose to spinal cord PRV (0.03 cc) | ≤50 Gy | ≥50 Gy but ≤52 Gy | >52 Gy |

All dose constraints to critical structures include the volume of the critical structure outside of the planning target volume. Standard dose constraints were adapted from RTOG 1016 protocol (NCT01302834).

Spinal cord: 0.03 cc of the PRV should not exceed ≥50 Gy. 0.03 cc of the spinal cord should not exceed ≥45 Gy.

Brainstem: 0.03 cc of the brainstem should not exceed 60 Gy. 0.03 cc of the PRV brainstem should not exceed 63 Gy.

Lips: Reduce dose as much as possible, with goal of mean dose <20 Gy.

Oral Cavity: Reduce dose as much as possible, with goal of mean dose <30 Gy for the uninvolved oral cavity. Hot spots >60 Gy should be avoided as possible within the uninvolved oral cavity.

Parotid Glands: Each parotid gland should be optimized separately, with a goal of mean dose <26 Gy.

Contralateral Submandibular Glands: If contralateral nodal level IB is not targeted, goal is to reduce mean contralateral submandibular to <39 Gy.

OARpharynx: Reduce the dose as much as possible with goal mean dose <45 Gy.

Esophagus: Reduce the dose as much as possible, with goal mean dose <30 Gy.

Supraglottis: Reduce the dose as much as possible, with goal mean dose <45 Gy.

Larynx: Reduce the dose as much as possible, with goal mean dose <45 Gy.

GSL: Reduce the dose as much as possible, with goal mean dose <45 Gy.

**Appendix 2.** Therapist Delivery Treatment Timeout

| Two therapists must be present for the treatment timeout.    1. Current practices of verifying patient and treatment site must occur.    2. The treatment navigator in Mosaiq must be used to confirm the treatment plan fraction delivered the day before and determine the next appropriate fraction. Fractions will be delivered in a pattern of Plan A – Plan B – Plan C – Plan D – Plan E. For example, if plan B was delivered the day prior (i.e. on a Wednesday) then plan C must be delivered next for the next fraction (i.e. Thursday). In this example, Plan D would be delivered on Friday and Plan E would be delivered on Monday.  · In the event the patient misses a planned treatment fraction, the therapist must notify specified physician and physicist. The determination can then be made in how to update the patient data in Mosaiq. Treatments should be resumed as soon as possible. The patient should resume therapy following the same pattern previously used A-B-C-D-E. Missed fractions should NOT be added to the end of the treatment schedule. Two fractions can never be delivered in the same day.  · No overrides are allowed to occur without the presence of a physicist. |
| --- |
